# What Can Neuroscience Tell Us about the Hard Problem of Consciousness?

**DOI:** 10.3389/fnins.2016.00395

**Published:** 2016-09-07

**Authors:** Berit Brogaard, Dimitria Electra Gatzia

**Affiliations:** ^1^Brogaard Lab for Multisensory Research, University of MiamiCoral Gables, FL, USA; ^2^Department of Philosophy, University of OsloOslo, Norway; ^3^Department of Philosophy, University of Akron Wayne CollegeOrrville, OH, USA

**Keywords:** consciousness, paradigm, psychotropics, schizophrenia, metastability, neuroimaging

A main research goal within neuroscience is to explain the relation between neurophysiological processes and conscious experiences. One approach involves focusing on problems such as the integration of information, the deliberate control of behavior, the ability to discriminate and categorize environmental stimuli, etc. These problems have been dubbed by philosophers as “easy” to suggest that the present limitations hindering progress could be overcome by more sophisticated methods in the near future (Chalmers, 1995, 1996). For example, explaining the integration of information requires describing the neurophysiological mechanisms responsible for information processing. Although these mechanisms are not currently well known, it is very likely that neuroscience will be able to explain them in the near future. It has been argued, however, that the problem of phenomenal consciousness cannot be explained by reference to such mechanisms because it involves a special kind of subjective qualities, i.e., phenomenal qualities that are present in experience (Dennett, 1991, 2003; Block, 1995; Chalmers, 1996; Revonsuo, 2006; Majeed, 2016). For example, the experience of seeing a red patch has a different phenomenal quality (a redly quality or “feel”) from the experience of seeing a green patch (a greenly quality or “feel”)^1^. The problem of explaining how or why neurophysiological processing gives rise to phenomenal experiences has been dubbed the “hard problem of consciousness” to suggest that solving it requires a paradigm shift in neuroscience (Chalmers, 1995, 1996).

There are several theoretical frameworks which attempt to integrate brain and mind (Crick and Koch, 1990, 2003; Edelman and Tononi, 2000; Revonsuo, 2000, 2003; Baars, 2003; Zeki, 2003, 2004; Freeman, 2007). However, most of them attempt to explain phenomenal consciousness through its neural correlates (Rees et al., 2002; Noë and Thompson, 2004). At the core of these theoretical frameworks is the idea that by isolating the physical processes associated with conscious experiences we can find comprehensive, systematic associations between them and characteristics of conscious experience. According to one such theory, phenomenal consciousness arises from a correspondence between the structural properties of the information processed in the brain and structural properties of conscious experience (see for example, Kohler, 1947). According to another such theory, consciousness arises from synchronized oscillations in the cerebral cortex (Crick and Koch, 1995). However, these theories have several drawbacks (Fingelkurts et al., 2009). Most notably, they rely on the relation of correlation, which is too weak to have any explanatory power (Chalmers, 1997; Noë and Thompson, 2004; Revonsuo, 2006; Fingelkurts et al., 2009) or postulate entities that cannot be easily measured scientifically (Revonsuo, 2000; Seth et al., 2008).

An alternative theoretical framework, known as Operational Architectonics, has recently been proposed to explain phenomenal consciousness (Fingelkurts et al., 2009, 2010). It does not reduce phenomenal experience to the brain functions since it is believed that such a reduction would either distort or eliminate consciousness. Rather, it purports to establish that the level of organization in the cortex is functionally isomorphic to the phenomenal level of experience. In other words, the phenomenological structure of consciousness corresponds to the brain's structure of operational architectonics (Fingelkurts and Fingelkurts, 2001; Fingelkurts et al., 2010). On this view, the cortical system can perform many operations, involving large-scale local and global cortical networks, characterized by different spatial and temporal parameters, using one or more operational modules. These operational modules are not directly connected with structural or static functional modules in the brain. Rather, they have a dynamic nature–they are metastable–and exist in their own operational space-time, i.e., an abstract space and time the brain constructs each time an operational module emerges (Fingelkurts and Fingelkurts, 2001; Fingelkurts et al., 2009, 2010)^2^. These metastable brain states underlie complex brain functions and corresponding conscious complex operations such as cognitive percepts and mental states that are representational in nature (Fingelkurts and Fingelkurts, 2001).

Rapid advances in the field of neuroimaging techniques including magnetoencephalography (MEG), electroencephalography (EEG), functional MRI (fMRI), diffusion tensor imaging (DTI), voxel based morphomentry (VBM), and optical imaging, have allowed neuroscientists to investigate neurophysiological processes in ways that have not been possible until recently (Tehovnik et al., 2006; Bandettini, 2009). Combining these techniques with advanced analysis procedures during different conditions such as hypnosis, psychiatric and neurological conditions, subliminal stimulation, and psychotropic drugs (Bhagwagar et al., 2006; Griffiths et al., 2008; Studerus et al., 2011; Lee and Roth, 2012) began transforming the study of neuroscience in ways that it is now possible to test such frameworks, bringing us a step closer to solving the hard problem of consciousness.

A study using a task-free functional MRI (fMRI) protocol designed to capture the transition from normal waking consciousness to the psychedelic state and blood-oxygen level-dependent (BOLD) fMRI to map cerebral blood flow and changes in venous oxygenation before and after intravenous infusions of placebo and psilocybin found that psilocybin significantly decreases the positive coupling of medial prefrontal cortex and posterior cingulate cortex (two key structural hubs) (Carhart-Harris et al., 2012; see also Lee and Roth, 2012)^3^. Although, the function of the posterior cingulate cortex is not yet fully understood, its association with the default-mode network regions (Raichle et al., 2001), known to host the highest number of cortico-cortical connections in the brain, suggests that it may play a role in conscious experience (Raichle, 1998; Gusnard et al., 2001) and may account for findings indicating that functional decreases in activity in the posterior cingulate cortex alter the conscious states of subjects under the influence of psilocybin (Carhart-Harris et al., 2012; Wood et al., 2012).

Neuroimaging techniques also indicate that psychotropic drugs can modify the natural metastable organization of brain activity (Fingelkurts et al., 2009). For example, a study in which healthy subjects were administered lorazepam (a benzodiazepine) showed that the number and strength of functional cortical connections increased significantly. These cortical changes were found to correspond to mental changes such as slowing of thinking and cognition (Fingelkurts et al., 2004). Such findings are consistent with the Operational Architecture framework, which posits that changes at mental level must be accompanied by corresponding changes in the metastable organization of brain activity (Fingelkurts et al., 2009).

A number of studies using neuroimaging techniques have also begun to examine the neural correlates of the phenomenal characteristics associated with auditory hallucinations in schizophrenic patients. Reduction in the prefrontal functional connectivity was found in studies using functional magnetic resonance imaging (fMRI) on eight schizophrenia patients and 10 controls in schizophrenia suffering from auditory hallucinations (Lawrie et al., 2002; see also Jardri et al., 2011; Allen et al., 2012). Although, more studies are needed, schizophrenia can provide an alternative approach to studying the functional connectivity of the brain and its relation to the resulting altered phenomenal states. For example, it is suggested that the altered phenomenal states in schizophrenia may be the result of a disruption of the metastable balance between large-scale integration and independent processing in the cortex, leading to an excess of local information expression by cortical areas (Bressler, 2003; Fingelkurts et al., 2009). Such suggestions could be tested in future studies.

The recent paradigm shift in neuroscience, which involves testing competing theoretical frameworks using a combination of neuroimaging techniques and advanced analysis procedures during different conditions, may allow us to find an adequate solution to the hard problem of consciousness. For example, the Operational Architecture framework posits that every change in the mental level must be accompanied by a corresponding change at the neurophysiological level (Fingelkurts et al., 2009). Unlike other frameworks, which rely on the relation of correlation that is too weak to have any explanatory power, this framework provides scientifically plausible ways to investigate the hard problem of consciousness. Different conditions such as psychotropic drugs, hypnosis, and schizophrenia can be used to test the hypothesis that mental changes must be accompanied by corresponding changes at the neurophysiological level. At the same time, neuroimaging techniques such as fMRI or EEG can be used to test whether changes at the neurophysiological level involve metastable changes between different functional operational modules in the cortex. It should nevertheless be emphasized that research on the hard problem of consciousness is currently in its infancy as a result of its perplexing nature. Although, the current paradigm shift discussed here may better prepare researchers to tackle the hard problem of consciousness, a considerable amount of research is required in order to arrive at any firm conclusions.

## Author contributions

All authors listed, have made an equal, substantial, direct and intellectual contribution to the work, their names appear alphabetically, and approved it for publication.

### Conflict of interest statement

The authors declare that the research was conducted in the absence of any commercial or financial relationships that could be construed as a potential conflict of interest.

## Footnotes

^1^The presence of an external stimulus is not necessary for having a conscious experience with a certain phenomenal quality; hallucinating a pink elephant involves having a conscious experience with a certain phenomenal quality in the absence of a sensory stimulus.

^2^Metastability initiates the hierarchical coupling between phenomenal and neurophysiological states while simultaneously allowing them to retain their individuality (Fingelkurts et al., 2009). It expresses the full complexity of a system, accommodates heterogeneous elements, does not require disengagement mechanisms in order for a system to switch to another state, and allows a system to flexibly survey through a set of possibilities (see Kelso and Tognoli, 2007).

^3^For studies using varying neuroimaging techniques such as arterial spin labeling (ASL), blood oxygen level-dependent (BOLD) measures, magnetoencephalography (MEG), or electroencephalography (EEG) and varying psychotropic substances such as LSD and ayahuasca (see Riba et al., 2002; Stuckey et al., 2005; Carhart-Harris et al., 2016).
